# Radiocarpal rotatory subluxation associated with a small radial styloid/rim avulsion after a bicycle fall: a case report

**DOI:** 10.1177/17531934261430179

**Published:** 2026-03-12

**Authors:** Max M Lokhorst, Jan Debeij

**Affiliations:** 1 Department of Plastic, Reconstructive and Hand Surgery, HagaZiekenhuis, The Netherlands; 2 Department of Plastic, Reconstructive and Hand Surgery, Amsterdam UMC, The Netherlands

**Keywords:** Carpal subluxation, distal radius fracture, diagnostic imaging, radial rim fracture, rotatory subluxation, surgical treatment, wrist injury

## Abstract

A case of anterior radiocarpal rotatory subluxation after wrist trauma highlights the need for further imaging when clinical symptoms are severe despite subtle initial radiographic findings. Timely surgical fixation led to full recovery and restored hand function at 3 months.

**Level of evidence:** V

A 37-year-old woman fell from her bicycle onto her extended left wrist, presenting to the emergency department with pain at the distal radius, swelling and impaired function without paraesthesia. Initial radiographs were reported as showing no distal radial or ulnar fractures (Figure 1). In retrospect, a small radial styloid/rim avulsion fragment could be seen. She was treated with a forearm cast and scheduled for review.

**Figure 1.**
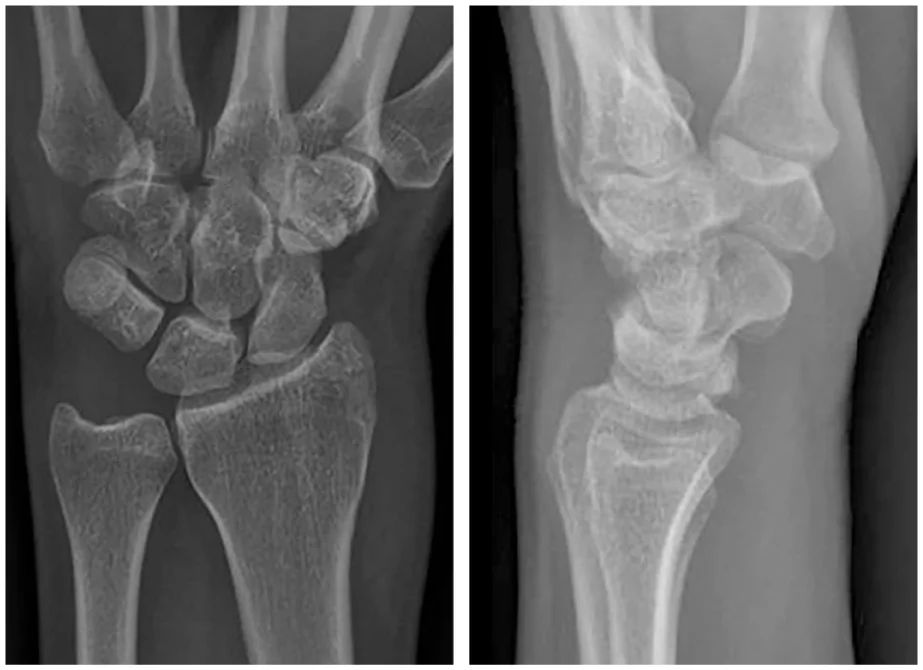
Initial posteroanterior and lateral radiographs of the left wrist. In retrospect, a small radial styloid/rim avulsion fragment is visible; radiocarpal incongruity is subtle on the lateral view.

At 1 week follow-up, swelling and ecchymosis were pronounced and out of proportion to the minor radiographic findings. Careful reassessment of the lateral radiograph suggested subtle radiocarpal incongruity. A CT scan was obtained to clarify the injury pattern and showed a small avulsed radial styloid/rim fragment with displacement and associated radiocarpal rotatory subluxation (Figure 2). Given the instability, fixation of the radial fragment was indicated.

**Figure 2.**
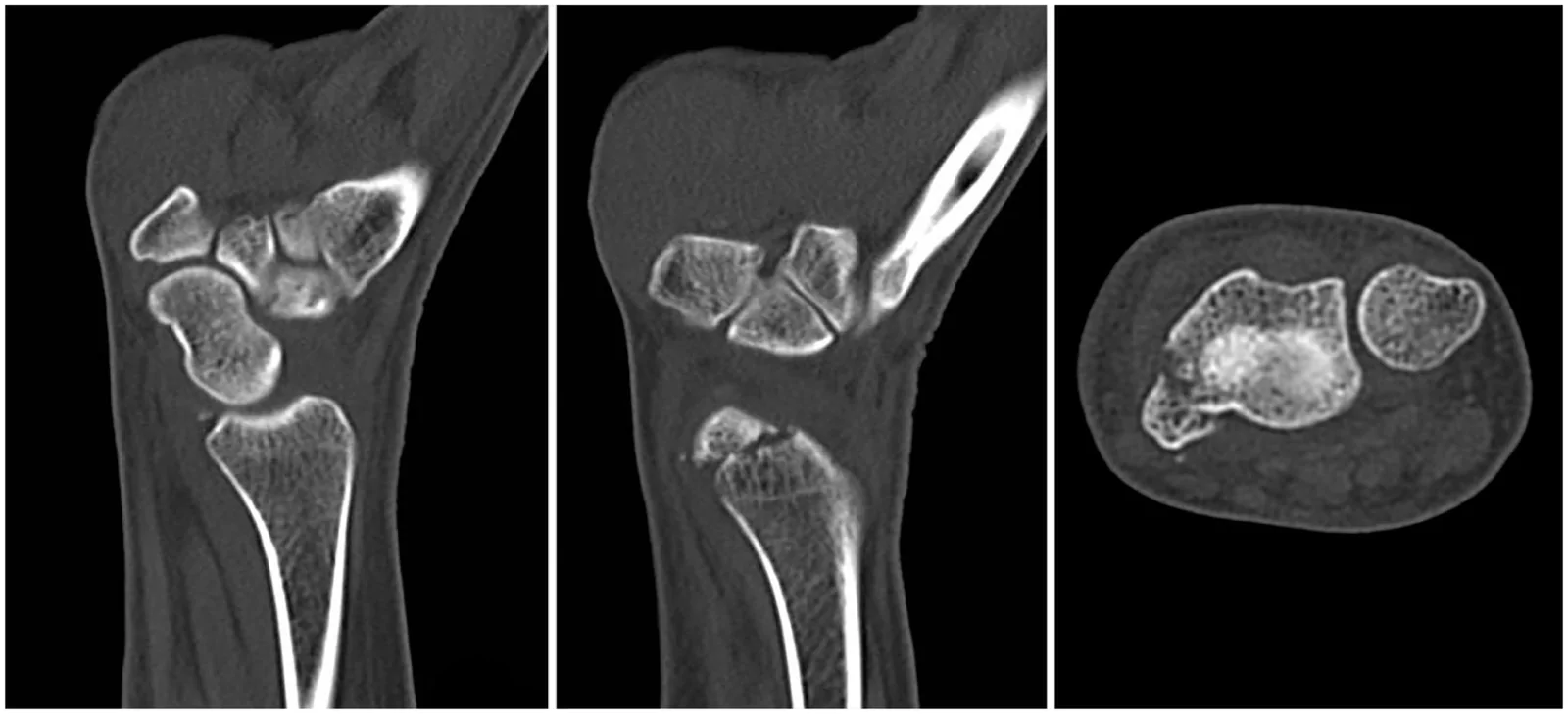
CT showing the small avulsed radial styloid/rim fragment with displacement and associated radiocarpal rotatory subluxation.

The fracture was reduced through a mini-open approach distal to the first extensor compartment and fixed with a single headless Mini-Acutrak compression screw (Acumed, Hillsboro, OR, USA) (Figure 3). A pressure garment was applied for 2 days; the patient was instructed to avoid loading the wrist and started hand therapy. At 3 months, there was full wrist and hand function. Grip strength measured with a dynamometer was comparable with the contralateral side. CT confirmed fracture union and restoration of radiocarpal congruity.

**Figure 3.**
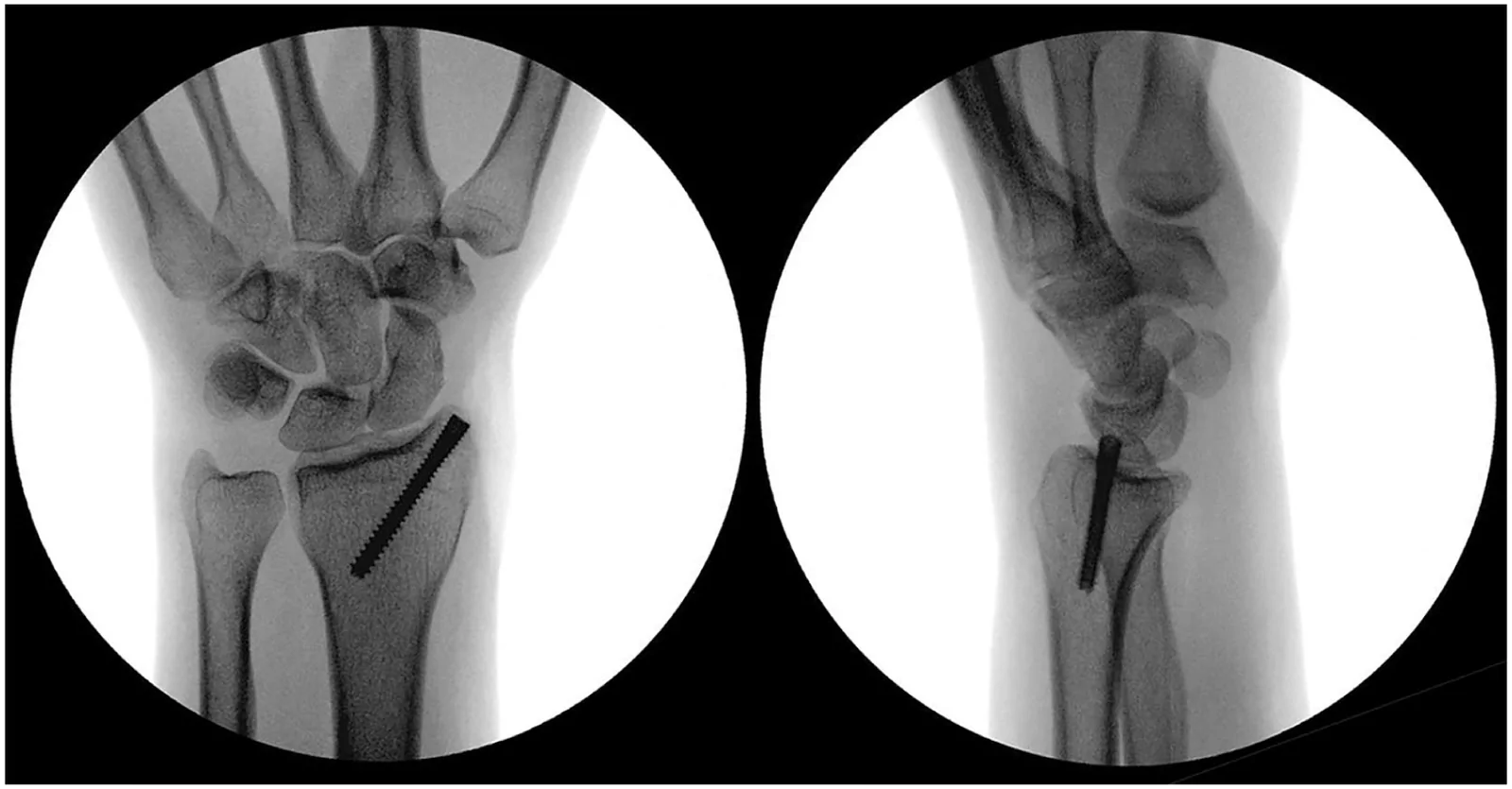
Intraoperative fluoroscopy after mini-open reduction and fixation with a single headless compression screw.

Radiocarpal subluxation is uncommon and can be missed on initial radiographs, particularly when the osseous component is small (Dumontier et al., 2001; Paula et al., 2022). The key learning point in this case is that a small radial rim/styloid fragment may represent a radiocarpal rim avulsion with a disproportionate destabilizing effect, leading to rotatory subluxation. Such injuries may be considered within the spectrum of complex carpal fracture-dislocation patterns, including inferior arc involvement (Graham, 2003).

When clinical findings are severe despite subtle radiographic abnormalities, careful assessment of carpal alignment on the lateral view is essential, and CT should be considered. In this case, CT was instrumental in identifying the rotational component and defining the fracture morphology. The case is presented because it illustrates a common pitfall: an apparently minor-appearing rim/styloid fragment on radiographs may coexist with clinically significant radiocarpal instability (Anderson and Meals, 2020). Prompt recognition and stabilization, followed by appropriate rehabilitation, can restore congruity and enable rapid functional recovery.
